# The Effects of Benzodiazepine Use and Abuse on Cognition in the Elders: A Systematic Review and Meta-Analysis of Comparative Studies

**DOI:** 10.3389/fpsyt.2020.00755

**Published:** 2020-09-17

**Authors:** Linzi Liu, Linna Jia, Peiying Jian, Yifang Zhou, Jian Zhou, Feng Wu, Yanqing Tang

**Affiliations:** ^1^ Department of Psychiatry, The First Affiliated Hospital, China Medical University, Shenyang, China; ^2^ Department of Psychology, Queen’s University, Kingston, ON, Canada; ^3^ Department of Geriatrics, The First Affiliated Hospital, China Medical University, Shenyang, China

**Keywords:** benzodiazepines, cognitive function, substance addiction, aged, meta-analysis, cognitive dysfuction

## Abstract

**Objective:**

Benzodiazepines (BZD) are one of the most frequently prescribed drugs worldwide. However, the cognitive effects of benzodiazepines in the elderly are highly debated. This systematic review and meta-analysis aims to explore the following two questions in the elderly population: (i) Do BZD lead to any impairments in cognitive functions in elderly users? and (ii) Which specific cognitive domains are most affected by BZD use and abuse?

**Methods:**

First, we performed a literature search following the PRISMA guidelines. Electronic databases, including PubMed, PsycINFO, EMBASE, Cochrane Library, and Web of Science were searched until May 14^th^, 2020. After selecting the relevant articles, we integrated the results of the selected studies with a standardized cognitive classification method. Next, we performed meta-analyses with the random-effects model on the cognitive results. Finally, we specifically examined the cognitive impairments of BZD in the abuse subgroup.

**Results:**

Of the included studies, eight of the thirteen had meta-analyzable data. Compared to the controls, elderly BZD users had significantly lower digital symbol test scores (n=253; SMD: -0.61, 95% CI: -0.91 to 0.31, I² = 0%, p < 0.0001). There was no significant difference in Mini-Mental State Examination, Auditory Verbal Learning Test, and Stroop Color and Word Test scores between BZD users and controls. According to the subgroup analyses, BZD abusers performed significantly worse than controls in Mini-Mental State Examination (n=7726; SMD: -0.23, 95% CI: -0.44 to -0.03, I² = 86%, p = 0.02), while there was no significant difference between the regular BZD users and the controls (n=1536; SMD: -0.05, 95% CI: -0.59 to 0.48, I² = 92%, p =0.85).

**Conclusion:**

In the elderly population, the processing speed (digital symbol test scores) was significantly impaired in BZD users; global cognition (Mini-Mental State Examination scores) was significantly impaired in BZD abusers but not in BZD regular users. This study provides insight into the factors that interact with BZD cognitive effects, such as aging, testing tools, and abuse. Clinicians should be cautious when prescribing BZD for the elderly.

**Systematic Review Registration:**

PROSPERO, identifier CRD42019124711.

## Introduction

Benzodiazepines (BZD) are two-ring heterocyclic compounds consisting of a benzene ring fused with a diazepine ring. Since its discovery in the 1950s, BZD’s sedative, hypnotic, anti-anxiety, and anti-convulsive effects have been increasingly accepted, making BZD use highly prevalent among adults (1) and especially in the elders (2). The prevalence of BZD use in elders varies between 10% and 42% worldwide (3). For example, BZD and related drugs are the third most abused prescription drug in America, with roughly 1-3% of the world population being subject to abuse (4). However, inappropriate BZD prescriptions can promote BZD misuse, facilitate the development of BZD addiction, and significantly affect the users’ overall quality of life (5, 6). Therefore, it is critical for pharmacists, clinicians, and patients to be informed on the latest research regarding the adverse effects of BZD use and abuse.

Since the 1970s, research has found negative effects of BZD on recipients’ cognitive functions (7). A meta-analysis in 2017 investigated the long-term cognitive impacts of BZD among adults. This analysis reported impairments in working memory, language, and processing speed, but not in executive function (reasoning and planning) (8). The participants in the meta-analysis, however, included adults of all ages. Compared to young adults, elderly populations require more cautiousness when undertaking BZD therapy. The elderly are more susceptible to cognitive impairment than young adults (9). During the progress of aging, cognitive function continues to decline with structural and functional neurological changes (10, 11). The pharmacokinetics of BZD in the elderly are different than in young adults, so the effects of BZD in older adults may have unique characteristics than in any other age group (12).

In recent years, several systematic reviews have found that BZD use was significantly associated with a higher risk of dementia and mild cognitive impairments (MCI). Dementia and MCI introduced a significant growth in mortality and financial burdens worldwide (13, 14). The most prominent symptom of these neurological disorders is a decline in cognitive function, measured by tests such as the Mini-Mental State Examination (MMSE). Sufficient evidence has shown that the risk of dementia correlates with cumulative dose, treatment duration, and long-acting effects of BZD molecules (15–18). However, previous literature failed to provide a causal account for the link between BZD use and the risk of dementia (18). To understand the mechanism behind the adverse effects of BZD, it is crucial to investigate the specific areas of cognitive functions that are impacted by BZD use and abuse.

Literature in the past 50 years presents contradicting evidence on whether BZD impairs cognitive functioning in the elderly (19–24). Among the studies that suggest an association between BZD use and cognitive impairment in elders, the type and degree of cognitive impairment reported are inconsistent across studies (19, 20, 22). Moreover, the neurological alterations due to the long-term effects of BZD use remains unclear (9). In 2018, Picton and colleagues summarized the cognitive effects of BZD in the elderly from published evidence. They found mixed findings of the association between BZD therapy and cognitive decline in elderly users (25). Given the lack of consensus in the current literature, a meta-analysis study may help reveal the critical effects of BZD use in the elderly and identify areas that require further research. To our knowledge, there are no systematic reviews with a meta-analysis that summarizes the current status of the cognitive effect of BZD use and abuse in the elderly population.

This systematic review and meta-analysis aims to explore the following two questions in the elderly: (i) Is BZD use associated with impairment in cognitive functions in the elderly? and (ii) Which cognitive domains have declined functionality associated with BZD use and abuse? The answers could help characterize the specific cognition impairments associated with BZD use, and identify individuals vulnerable to the negative effects of BZD. This meta-analysis may also help identify and monitor the cognitive effects associated with BZD use and abuse to prevent BZD addiction. With the high prevalence of BZD being prescribed to older populations worldwide, it is essential to inform clinicians and patients about the possible cognitive impairments associated with BZD use and abuse. Given the refractory rate and adverse effects of dementia and MCI (26), the results may also help reduce inappropriate BZD prescriptions to attenuate dementia risk in the elderly population.

## Methods

### Systematic Review Protocol

The process of this systematic review follows the Preferred Reporting Items for Systematic Reviews and Meta-Analyses (PRISMA) guidelines, 2009 (27).

### Databases and Search Strategy

We searched relevant articles in electronic databases, PubMed, PsycINFO, EMBASE, Cochrane Library, and Web of Science, from their inception to May 14, 2020. Specific search keywords included “benzodiazepines”, “cognition”, and “aged”. The query used for PubMed is ((Benzodiazepines[mh]) OR (Benzodiazepines Compounds[Title/Abstract]) OR (Benzodiazep*[Title/Abstract])) AND (cognit *’ OR memory OR attention OR visual-spatial OR visuospatial OR recall OR recognition OR problem solving OR reaction time OR vigilance OR executive function*’ OR reasoning OR psychomotor OR motor OR processing OR planning OR verbal fluency OR inhibit *’) AND ((Aged[mh]) OR (Elder*[Title/Abstract]) OR (older adults[Title/Abstract])).

Additionally, we searched in Google Scholar and searched the reference list for relevant articles to ensure that no studies were missed.

### Inclusion Criteria and the Process to Identify Studies

We developed the inclusion criteria according to the PICOS guideline. Appropriate papers met the following criteria: 1) Participants were human adults, older than 60 years, mean sample age over 65 years; 2) the treatment groups were BZD users; 3) the studies had placebo or non-users of BZD as controls; 4) the outcomes included performances of cognitive functions measured using any standardized neuropsychological instruments; and 5) any type of published clinical studies except for case reports or conference abstracts were used. We only included articles reported in English.

Endnote was used to delete the duplicated articles. Two reviewers (LL and JL) independently scanned the references by title/abstract to exclude irrelevant articles, then read the full text to identify the appropriate studies based on the above inclusion criteria. Finally, the debated studies were determined through discussions with a third reviewer (YT).

### Risk of Bias and Quality Assessment

Two reviewers assessed the quality of each included study independently with the modified Newcastle-Ottawa Scale (mNOS) (28). The mNOS examines the quality of non-randomized studies by four bias reduction items: (1) selection bias, (2) performance bias, (3) detection bias, and (4) reporting bias. Each item is graded from 0 (low quality) to 3 (high quality) according to the example given for each risk level. Disagreements were resolved by discussions with a third reviewer (Y.T.). Publication bias was tested by using the funnel plot and Begg’s tests on the outcome which synthesizes more than five studies.

### Data Extraction

After generating a list of the included articles, two reviewers made the data extraction form collectively. The two reviewers then extracted the data independently. The data extraction form contained the following information: (1) author and publication year, (2) study design, (3) setting (country), (4) study design, (5) mean age, (6) gender distribution, (7) education, (8) participants sources, (9) details of BZD use (BZD types, using time, dosage, and the definition of BZD use), and (10) outcomes (cognitive task, cognitive domain, and main findings). The authors were contacted in order to obtain any missing data.

### Data Synthesis and Statistical Analyses

The included studies utilized a variety of psychometric measurements, rendering it challenging to produce generalizable and informative conclusions. In this paper, we organize cognitive domains based on a commonly used framework (29, 30). According to this framework, we summarized the outcomes of the cognitive task from each included study to include a balanced and comprehensive view. Due to the discrepancy in psychometric screening tools between studies, the meta-analysis would only synthesis the data from the same neuropsychological instruments, such as the MMSE and the Auditory Verbal Learning Test (AVLT).

The RevMan software (31) was used to perform the meta-analysis. We adopted the random-effects model in our meta-analysis because it is more conservative than the fixed-effects model. Standardized mean difference (SMD) with 95% confidence intervals (CIs) was used for continuous outcomes. The study’s heterogeneity was measured using I^2^. I^2^>50% indicated significant heterogeneity (32). The meta-analytic outcomes were two-tailed, with a significance level of 0.05. Based on BZD use information reported in each study, we divided all the BZD users into two categories: regular BZD users and BZD abusers. BZD regular use is an appropriate pattern of BZD use that follows the prescription instruction. BZD abuse behaviors include meeting sedative, hypnotic, and anxiolytic use disorder criteria according to the Diagnostic and Statistical Manual of Mental Disorders, Fifth Edition, and using a higher frequency or dose, or longer use time than prescribed, or without a prescription (1, 4, 33). We then conducted a subgroup analysis to explore the cognitive changes in BZD abusers and regular BZD users.

## Results

### Search Results and Studies Included

**Figure 1:**
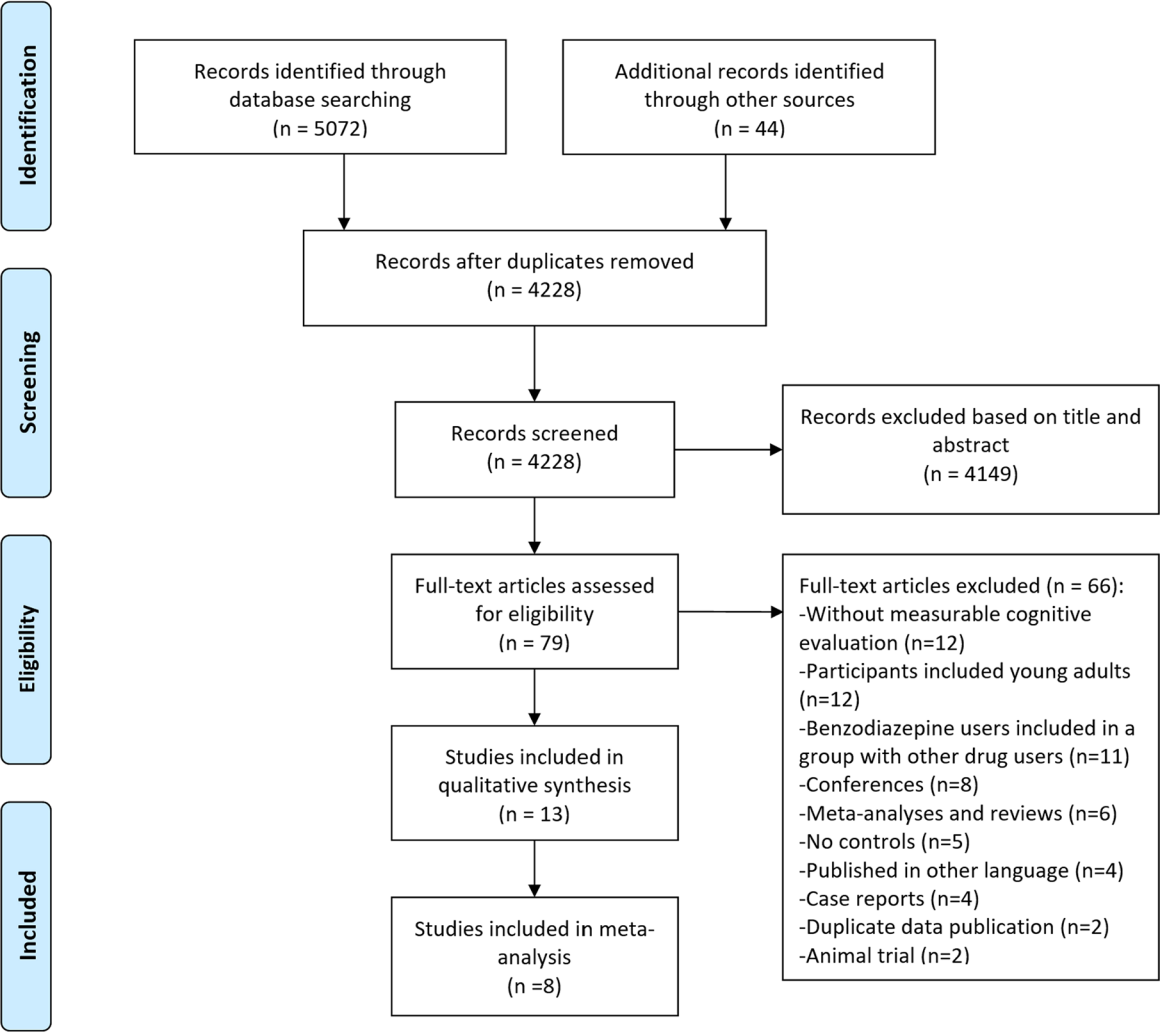
Flow diagram of the search and study identification process.

The study selection process of this systematic review is summarized in **Figure 1**. A total of 5072 references were returned by the initial search and scan, with 44 from relevant reviews and Google Scholar. After removing duplicated and irrelevant articles by title and abstract, the full texts of the remaining 79 were screened and 15 articles finally met the inclusion criteria. Two articles (34, 35) were from the same cohort study named *The Canadian Study of Health and Aging* (36). Another two articles had repeated participants (22, 37). Overall, 13 studies were included in the literature review. In the 13 studies, eight had meta-analyzable data.

### Risk of Bias and Quality Assessment

The risk bias of the included studies was assessed by the mNOS and detailed in **Table S1** in the **Supplementary Materials**. The scores ranged from 13-19 out of 21. Nine studies (34, 38–45) had a sample size of more than one thousand participants. Nine studies (34, 38, 40–42, 44–47) used instruments that measured multi cognition domains. Eleven studies matched or adjusted education level as covariates and 12 studies matched or adjusted age as covariates. Therefore, the overall risk bias of the included studies is reasonably low. We tested the publication bias of the meta-analysis results of MMSE scores; the funnel plot (**Figure S1** in **Supplementary Materials**) and Begg’s tests (p=0.673) did not find publication bias.

### Studies Characteristics

There were 26033 participants (6374 BZD users) included in this systematic review and 10666 (2318 BZD users) included in the meta-analysis. As shown in **Table 1**, all studies were carried out in America or Europe. Six out of thirteen were published in the last five years. Eight were cohort studies; the remaining five were cross-sectional studies. The mean age of the participants is 72.5 in the cohort studies at baseline, and 83.8 in the cross-sectional studies. Therefore, both users and controls included in this review were elderly participants. In the 13 studies, a total of 36 tasks were used to measure cognitive functions; each study utilized one to nine cognitive tasks.

**Table 1:** Summary of included articles.

Study	Study design	Country	Samples (BZD users)	Mean Age (BZD users)	Male percentage (BZD users)	Participants sources	Cognitive task	Cognitive domain
**Gray et al. (** 39 **)**	cohort study	U.S.	3434(1018)	74.4(74.5)	40.4(33.1)	population based	CASI	General Cognition
**Hanlon et al. (** 40 **)**	cohort study	U.S.	2765(400)	range(65-105)	33.5	community	SPMSQ; OMC	General Cognition
**Paterniti et al. (** 42 **)^a^**	cohort study	France	1176(159)	65(65.3)	41.6(23.7)	population based	TMT-B; DSS; RAVLT; FTT	General Cognition; Processing speed; Immediate recall: verbal/visual; Gross motor speed
**Bierman et al. (** 38 **)^b^**	cohort study	Netherlands	2105(1189)	69.2	47.5	population based	MMSE; Coding Task; RAVLT; RCPM	General Cognition; Processing speed; Immediate recall: verbal/visual; Reasoning/planning
**Mura et al. (** 41 **)^a,b^**	cohort study	France	5195(969)	73.5(74.6)	40.1(23.1)	community	MMSE; TMT-B; TMT-A; BVRT; IST	General Cognition; Processing speed; Immediate recall: verbal/visual; Verbal fluency
**Zhang et al. (** 43 **)^b^**	cohort study	U.S.	5423(405)	73.0(73.6)	34.1(30.6)	Alzheimer’s disease center	MMSE; CDR-SB	General Cognition
**Ros-Cucurull et al. (** 47 **)^a,b^**	cohort study	Spain	64(33)	73.2(73.5)	28.13(21.9)	BZD users from hospital; controls are volunteers	MMSE; CPT-II; SDMT; RCFT; CVLT; COWAT FAS; IGT; Tower of London Test; N-Back; SCWT	General Cognition; Vigilance/focus; Processing speed; Immediate recall: verbal/visual; Delayed recall: verbal/visual; Recognition: verbal/visual; Verbal fluency; Reasoning/planning; Working memory; Inhibitory control
**Del Ser et al. (** 44 **)**	cohort study	Spain	1087(810)	74.7	36.1	community	MMSE; FCSRT; semantic verbal fluency (animals in one minute); CDT; DST	General Cognition; Immediate recall: verbal/visual; Delayed recall: verbal/visual; Verbal fluency; Reasoning/planning; Processing speed
**Hoiseth et al. (** 46 **)^a^**	cross-sectional study	Norway	241(168)	78.6(78.1)	27.8(25.0)	hospital	MMSE; HVLT; SCWT; DVT	General Cognition; Vigilance/focus; Immediate recall: verbal/visual; Delayed recall: verbal/visual; Recognition: verbal/visual; Inhibitory control
**Helmes and Ostbye (** 34 **)^a^**	cross-sectional study	Canada	1754(408)	79.7(79.6)	38.7	community	WAIS Block Design; Buschke Free Recall; AVLT Trial 6; Verbal Fluency; Token Test; WAIS Information; WAIS Similarities; WAIS Comprehension	Processing speed; Immediate recall: verbal/visual; Recognition: verbal/visual; Fine motor speed; Verbal Fluency; Semantic processing; Reasoning/planning
**van Vliet et al. (** 45 **)^a,b^**	cross-sectional study	Netherlands	2275(702)	range(85-90)	28.0(20.0)	community	MMSE; LDT; PLT-I; PLT-d; SCWT the Third Chart	General Cognition; Processing speed; Immediate recall: verbal/visual; Delayed recall: verbal/visual; Inhibitory control
**Puustinen et al. (** 37 **)^a^**	cross-sectional study	Finland	119(64)	81.6(82.1)	23.53(15.6)	hospital	MMSE	General Cognition
**Hessmann et al. (** 48 **)^a,b^**	cross-sectional study	Germany	395(49)	78.8(84.0)	31.9(28.6)	hospital	MMSE	General Cognition

CASI, Cognitive Abilities Screening Instrument; SPMSQ, Short Portable Mental Status Questionnaire; OMC, Orientation-Memory-Concentration Test; MMSE, Mini Mental State Examination; TMT-B, The Trail Making Test, part B; DSS, The Digit Symbol Substitution Test; RAVLT, Rey Auditory Verbal Learning Test; FTT, Finger Tapping Test; RCPM, Raven’s Coloured Progressive Matrices; TMT-A, The Trail Making Test, part A; BVRT, The Benton Visual Retention Test; IST, Isaacs Set Test; CDR-SB, Clinical Dementia Rating Sum of Boxes; CPT-II, Conners Continuous Performance Test II-Omissions; SDMT, Symbol Digits Modalities Test; RCFT, Rey Complex Figure Test; CVLT, California Verbal Learning Test; COWAT FAS, Controlled Oral Word Association Test; IGT, Iowa Gambling Task; SCWT, Stroop Color and Word Test; FCSRT, total immediate and delayed recall in the Free and Cued Selective Reminding Test; CDT, clock drawing test; DST, digit symbol test; HVLT, Hopkins Verbal Learning Test; DVT, Digit Vigilance Test; LDT, Letter Digit Coding Test; PLT-I, Picture Learning Test-immediately; PLT-d, Picture Learning Test-delay.

Participants’ characteristics were collected according to baseline information.

^a^The authors report meta-analyzable data.

^b^The BZD abuse studies.

### Synthesized Findings

#### Benzodiazepine Use and Cognitive Decline in the Elders

**Figure 2:**
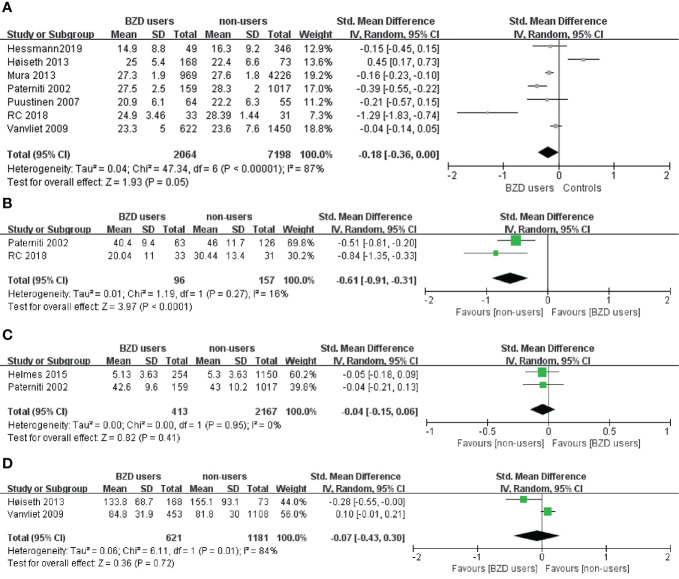
**(A)** Effect of BZD use on Mini Mental State Examination in the elderly: forest plot. **(B)** Effect of BZD use on Digital Symbol test in the elderly: forest plot. **(C)** Effect of BZD use on Auditory Verbal Learning Test in the elderly: forest plot. **(D)** Effect of BZD use on Stroop Color and Word Test in the elderly: forest plot.

**Table 2** provides an overview of the effects on cognitive function from BZD use consolidated from all the included studies. The classification cognitive function division was adapted from a commonly used method of understanding and measuring cognitive domains (29). Eight of the thirteen studies with cognitive performance data had meta-analyzable data. They used the same cognitive measurements and reported the quantitative results required for meta-analysis (**Table 1**). Our meta-analysis synthesized data obtained with the same cognitive tasks across the included studies. Overall, the meta-analysis included the following cognitive functions: global cognition measured with the MMSE, processing speed measured with the digital symbol test, memory (recognition) measured with the AVLT, and executive functions (inhibitory control) measured with the Stroop Color and Word Test (SCWT) (**Figures 2A–D**).

**Table 2:** Overview of tasks used to assess cognitive functioning in benzodiazepine users across different cognitive domains.

Cognitive domain	Task	Studies	Sensitivity^a^
**Attention and processing speed**			
**Vigilance/focus**	Conners Continuous Performance Test II-Omissions (CPT-II)	Ros-Cucurull et al. (47)*	1/1
	Digit Vigilance Test (DVT)	Hoiseth et al. (46)	0/1
**Processing speed**	TMT-B	Paterniti et al. (42); Mura et al. (41)*	2/2
	TMT-A	Mura et al. (41)*	1/1
	The Digit Symbol Substitution (DSS) test	Paterniti et al. (42)	1/1
	Coding task	Bierman et al. (38)*	0/1
	Symbol Digits Modalities Test (SDMT)	Ros-Cucurull et al. (47)*	1/1
	WAIS Block Design	Helmes and Ostbye (34)	0/1
	Letter Digit Coding Test (LDT)	van Vliet et al. (45)*	0/1
	Digit Symbol Test (DST)	Del Ser et al. (44)	1/1
			7/11
**Memory and learning**			
**Immediate recall: verbal/visual**	Rey Auditory Verbal Learning Test (RAVLT)	Paterniti et al. (42); Bierman et al. (38)	0/2
	The Benton Visual Retention Test (BVRT)	Mura et al. (41)*	1/1
	Rey Complex Figure Test (RCFT) Immediate recall	Ros-Cucurull et al. (47)*	1/1
	California Verbal Learning Test (CVLT)	Ros-Cucurull et al. (47)*	1/1
	Hopkins verbal learning test (HVLT)	Hoiseth et al. (46)	0/1
	Buschke free recall	Helmes and Ostbye (34)	1/1
	Picture Learning Test (PLT-i)	van Vliet et al. (45)*	0/1
	Free and Cued Selective Reminding Test (FCSRT)-immediate recall	Del Ser et al. (44)	1/1
			5/9
**Delayed recall: verbal/visual**	Rey Auditory Verbal Learning Test (RAVLT)	Paterniti et al. (42); Bierman et al. (38)*	1/2
	Rey Complex Figure Test (RCFT) Delayed recall	Ros-Cucurull et al. (47)*	1/1
	California Verbal Learning Test (CVLT)	Ros-Cucurull et al. (47)*	0/1
	Hopkins verbal learning test (HVLT)	Hoiseth et al. (46)	0/1
	Picture Learning Test (PLT-d)	van Vliet et al. (45)*	0/1
	the Orientation- Memory-Concentration Test (OMC)	hanlon1998* (40)	1/1
	Buschke free recall	Helmes and Ostbye (34)	1/1
	Free and Cued Selective Reminding Test (FCSRT)-delayed recall	Del Ser et al. (44)	0/1
			4/9
**Recognition: verbal/visual**	Rey Auditory Verbal Learning Test (RAVLT)	Paterniti et al. (42); Bierman et al. (38)*; Helmes and Ostbye (34)	1/3
	Rey Complex Figure Test (RCFT) recognition	Ros-Cucurull et al. (47)*	0/1
	California Verbal Learning Test (CVLT)	Ros-Cucurull et al. (47)*	0/1
	Hopkins Verbal Learning test (HVLT)	Hoiseth et al. (46)	0/1
			1/6
			9/23
**Motor**			
**Gross motor speed**	the Finger Tapping Test (FTT)	Paterniti et al. (42)	1/1
**Fine motor speed**	WAIS Block Design	Helmes and Ostbye (34)	0/1
			1/2
**Language**			
**Verbal fluency**	The Isaacs Set Test (IST)	Mura et al. (41)*	1/1
	Controlled Oral Word Association Test (COWAT FAS)	Ros-Cucurull et al. (47)*	1/1
	verbal fluency	Helmes and Ostbye (34); Del Ser et al. (44)	0/2
**Semantic processing**	Token Test	Helmes and Ostbye (34)	1/1
	WAIS Information	Helmes and Ostbye (34)	0/1
			3/6
**Executive functions**			
**Reasoning/planning**	Raven’s Colored Progressive Matrices (RCPM)	Bierman et al. (38)*	1/1
	Iowa Gambling Task(IGT)	Ros-Cucurull et al. (47)*	0/1
	Tower of London Test	Ros-Cucurull et al. (47)*	1/1
	WAIS Similarities	Helmes and Ostbye (34)	0/1
	WAIS Comprehension	Helmes and Ostbye (34)	1/1
	clock drawing test	Del Ser et al. (44)	0/1
**Working memory**	N-Back	Ros-Cucurull et al. (47)*	1/1
**Inhibitory control**	SCWT	Ros-Cucurull et al. (47)*; Hoiseth et al. (46)	1/2
	the third chart of the 40-item SCWT	van Vliet et al. (45)*	0/1
			5/10

* for BZD abuse studies.

Eleven out of 14 experiments on global cognition in the 13 studies showed no relationship between BZD use and decreased performance in global cognition. However, two experimental results (42, 47) showed that BZD users with higher socioeconomic status and BZD abusers performed worse in the MMSE than non-using controls. The remaining study by Bierman et al. (38) provided a third alternative result. The population-based 9-year cohort study concluded that, although there is a significant negative correlation between the MMSE scores and the accumulated BZD, the decrease in the MMSE scores with BZD use was relatively small. Moreover, the authors reported no correlation between dosage of BZD and MMSE scores. Taken together, the conclusions drawn from the literature are consistent with our meta-analysis results (**Figure 2A**); BZD users did not show significantly worse performance in the MMSE compared to the controls (n=9262; SMD: -0.18, 95% CI: -0.36 to 0.00, I² = 87%, p=0.05).

Seven studies tested the processing speed of BZD users. Four studies concluded that BZD use in the elderly population may result in significant impairment (41, 42, 47). They tested this domain mainly with the Trail Making Test (TMT) or the digit symbol test. The results showed that BZD users performed significantly worse across all the abovementioned tests than the controls. A meta-analysis (**Figure 2B**) was performed using the two studies that utilized the TMT. The results showed that in the elderly, the BZD users performed significantly worse than controls in digit symbol tests (n=253; SMD: -0.61, 95% CI: -0.91 to 0.31, I² = 0%, p < 0.0001). However, three other studies (34, 38, 45) suggested that the BZD users’ processing speed was not significantly impaired during a coding task and block design task.

An overview of the eight studies that tested memory and learning ability is presented in **Table 2**. Five out of nine tasks showed impairment in verbal/visual immediate recall among BZD users. More specifically, three out of five verbal immediate recall tasks and two out of four visual immediate recall tasks showed worse performances in BZD users than controls. When measuring with verbal/visual delayed recall tasks, four out of nine studies showed less accurate responses among BZD users. In these tasks, three out of seven verbal delayed recall tasks and one out of two visual delayed recall tasks showed BZD users performed worse than controls. The meta-analysis of the AVLT results (**Figure 2C**) is high in heterogeneity and non-significant (n=2580; SMD: -0.04, 95% CI: -0.15 to 0.06, I² = 84%, p=0.41).

Results of the tasks for motor, language ability, and executive functions are controversial (**Table 2**). In the domains of language ability and reasoning/planning function, three (34, 38, 47) out of four studies (44) that tested the respective domain showed a significantly worse performance in BZD users. Only one paper tested working memory (47) and showed that BZD users had a significantly worse performance than controls. In the domain of inhibitory control, the SCWT data sourced from two studies can be used for meta-analysis (**Figure 2D**). The results were non-significant and had high heterogeneity (n=1802; SMD: -0.07, 95% CI: -0.43 to 0.30, I² = 84%, p =0.72).

#### Benzodiazepine Abuse and Cognitive Decline in the Elders

**Figure 3:**
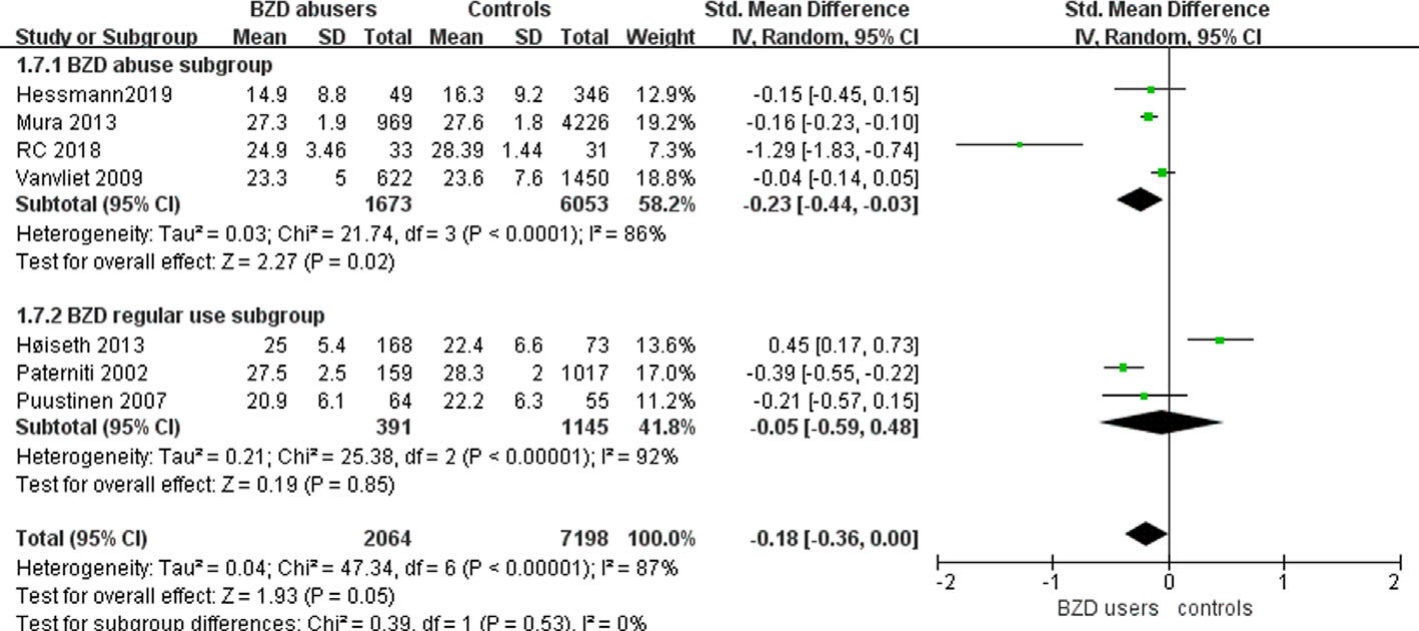
Subgroup analysis of the effect of BZD abuse and regular use on mini mental state examination in the elderly.

We conducted subgroup analyses of BZD abusers (**Figure 3**). Seven studies included participants with BZD abuse (38, 40, 41, 43, 45, 47, 48) (**Table 1**). In the BZD abuse subgroup, the abusers received significantly lower MMSE scores compared to the controls (n=7726; SMD: -0.23, 95% CI: -0.44 to -0.03, I² = 86%, p =0.02). Meanwhile, in the BZD regular use subgroup, the MMSE scores of BZD users versus controls were not significantly different (n=1536; SMD: -0.05, 95% CI: -0.59 to 0.48, I² = 92%, p =0.85). Considering I² >50%, the heterogeneity between the studies cannot be ignored.

Due to the discrepancy in the type of cognitive tasks used in the included studies, we could not obtain meta-analyzable data to examine the effects of BZD abuse on specific cognitive functions. Nevertheless, the results of the experiments in these studies were summarized in **Table 2**. Notably, for language ability, Mura et al. used the Isaacs Set Test to explore the effect of BZD abuse (41), and Ros-Cucurull used the Controlled Oral Word Association Test (47). Both results showed that BZD abusers performed worse than the control groups. In the domain of recognition, BZD abusers performed worse than controls in three different tasks in two studies (38, 47). Experiments testing performance on other cognitive divisions showed mixed results.

## Discussion

### Main Findings of BZD Use

This meta-analysis and systematic review included 6374 BZD users and 19,659 controls to comprehensively investigate the affected cognition domains by BZD use and abuse in elders. A total of 13 papers met the inclusion criteria and were included in the literature review. Eight out of the 13 papers had appropriate tests and sufficient information for the meta-analysis.

Consistent with previous systematic reviews, our meta-analysis suggests no impairment in global cognition among elderly BZD users (49, 50). Interestingly, studies with young adults showed opposing results (51, 52), suggesting BZD use significantly impairs participants’ global cognitive functioning. One reason for these results may be the fact that the negative effect of various risk factors on global cognitive decline decreases with age (53).

The results consistently showed impairment in elderly BZD users’ processing speed, but not inhibitory control. Processing speed, defined by the time it takes for a person to complete a mental task, has been found to be associated with caudate activity in neuroimaging studies (54). Meanwhile, a higher BZD dose is associated with volume reductions in the caudate nucleus (55). This imaging evidence supports our results of decreased processing speed among BZD users. Our result in memory functions, however, is inconsistent with previous studies. While the meta-analysis (8) with adults of all ages found that BZD users had a significantly impaired working memory and immediate memory ability, our systematic review and meta-analysis did not find sufficient support for the effect. However, the interaction between age and BZD’s effect on memory requires more robust validation.

### Main Findings of BZD Abuse

The BZD abuse subgroup in this review included 1673 BZD abusers and 6053 controls. The subgroup analysis showed that BZD abusers received significantly lower MMSE scores than the controls, while the BZD regular users’ scores were not significantly different from controls. These results demonstrated that impairment in global cognition occurs after the BZD user develops into abuse. However, the high heterogeneity in the results cannot be ignored. According to the results of the subgroup analysis, the confidence intervals of the two subgroups overlapped, and the difference between the two groups was not statistically significant (56). However, the subgroup analyses results, especially when not pre-specified at the beginning of the trial, cannot reflect the differences between the effect of interventions in subgroups (57). Future studies that directly contrast the cognition of BZD users and abusers are necessary. Only two studies tested language ability in BZD abusers; both found significantly worse language performance in BZD abusers compared to controls. Meanwhile, only two studies tested recognition ability; neither found a significant difference in the performance between BZD abusers and controls. These findings, however, are not conclusive because of the heterogeneity in the statistical results, and the small number of studies in the analyses. Therefore, more experiments are needed to reach more reliable conclusions.

After searching the databases, we did not find a meta-analysis investigating the cognitive effects of BZD abuse. The most relevant studies are two systematic reviews and meta-analyses papers on dementia risk in the elderly with long-duration and high dosage BZD users (58, 59). In 2015, Zhong and colleagues summarized six nested case-control or prospective cohort studies and concluded that higher dosage BZD users had an increased risk of dementia (59). In 2019, He et al. found that the risk of dementia was higher in patients taking BZD for a longer duration (>3 years) among six case-control and four cohort studies (58). Although long-term or high dosage use is not equivalent to dependence, BZD addiction is more likely to occur in long-term and high-dose users (60). These studies support the results of our subgroup analysis to some extent, but the mechanism and effects behind BZD addiction and abuse needs further investigation.

### Strengths and Limitations of This Review

This study is, to our knowledge, the first systematic review and meta-analysis of the cognitive effects of BZD use and abuse in elders. We attempted to consolidate results from studies testing different cognitive functions and data from a variety of cognitive tasks by utilizing a mature cognitive domain classification catalogue. The subgroup analysis of BZD abusers allowed us to preliminarily compare the effects of BZD use and abuse. This analysis encourages further studies to examine the qualitative difference of BZD use and abuse. These results can help identify and monitor the cognitive effects of BZD use and abuse, shedding light on awareness and prevention of BZD addiction at early stages, providing evidence for clinical decision-making, and improving the life quality of the elderly.

There are some limitations to this meta-analysis and systematic review. First, the number of studies and cognitive domains examined in this meta-analysis was limited. Five of the thirteen studies did not report cognitive tests data, and the domains of motor and language did not have meta-analysis results. Second, although the n is quite large, the meta-analysis results of the digital symbol test, AVLT, and SCWT were drawn from two small sample size studies, which is difficult to justify or interpret, considering the biased nature of the original studies. In the subgroup analysis of MMSE, the sample size of studies in the BZD regular users’ subgroup varied from hundreds to 2500, which are much smaller than the sample size of the abusers’ subgroup (>7000). Although the random effects model was used to reduce the impact caused by the difference between the sample size of the two subgroups, there was no analyzable data for further analysis. Third, due to the limitation of meta-analysis methodology, we could not directly compare the cognitive differences between BZD users and abusers. Fourth, other variabilities existed in the 13 studies in terms of the participants’ source (hospital or community), sample size, sex ratio, and cognitive measurements. These factors could also bias the findings to an uncertain extent. Fifth, users and controls were not well matched in the analysis. Participants in the experimental groups of the studies had different preexisting conditions such as anxiety, depression, insomnia, and APOE e4 status. One study did not age match the intervention group with the control group or as a covariable, which means the study could not completely distinguish the cognition impaired by BZD and by natural aging. Other factors related to cognition, such as the use of other psychotropic medications, certain physical diseases, and educational level, were not examined in most studies. Finally, due to the limited number of studies, sources of heterogeneity were not examined.

### Implications and Future Directions

Our meta-analysis confirmed the negative effects of BZD on elderly users’ processing speed. Therefore, doctors should be cautious when prescribing BZD drugs to elderly patients, especially those with family histories of dementia, Alzheimer’s disease, and other aging-related cognitive deficits. Additionally, although global cognition was not impaired in BZD regular users, BZD abusers had significantly worse performance in global cognition. This research can inform more individualized prescription decisions. For example, elderly patients whose daily activities require higher cognitive processing should be informed of BZD’s potential side effects on their cognitive processing speed. Patients with a history of addiction should prioritize alternative treatments to BZD therapy to prevent BZD dependence and abuse.

Another important finding in this study is that the results of cognitive performance are highly dependent on the type of cognitive measurements in the study. For example, as previously reported, BZD users had significantly lower processing speeds when tested with the TMT. However, studies measuring processing speed with the coding task or block design task did not reveal any significant findings. Therefore, clinical practitioners should be mindful when selecting cognitive tests. It might be reasonable to use tests with higher sensitivity to reduce missed diagnoses.

In addition, through our exploration in the literature on the cognitive effects of BZD use, few studies paid attention to BZD abuse and addiction in participants (47, 61, 62). BZD use and abuse can be qualitatively different from BZD use. A survey conducted in 2015-2016 showed that BZD abusers accounted for about 17% of BZD users (6). Moreover, approximately half of the patients who used BZD for longer than 1 month are subject to BZD abuse or addiction (60). The neglect of the BZD abuse subgroup may be accountable for the mixed conclusions from studies on the cognitive effects of BZD use. Therefore, we encourage future researchers to separate BZD regular users and BZD abusers to achieve more precise and rigid conclusions.

## Conclusions

In conclusion, this meta-analysis indicated no significant global cognition deficit (MMSE scores) in BZD users, but did reveal deficits in elders with BZD abuse behaviors. BZD users performed significantly worse in the cognition domain of processing speed (digit symbol test scores) than the controls, but not in memory and learning (AVLT scores) or inhibitory control (SCWT scores). Studies that tested the other cognitive domains, however, showed conflicting results. Unfortunately, these cognitive domains’ measurements varied across studies, rendering it unavailable to be merged into meta-analysis. Clinicians should be cautious when prescribing BZD for the elderly, especially to patients with a family history of age-related cognitive deficits. Moreover, the majority of the included studies did not clearly distinguish between the use and abuse of BZD, making it challenging to evaluate the effects of BZD abuse. Future well-designed studies are needed in order to verify the cognitive effects of BZD use and abuse.

## Data Availability Statement

The raw data supporting the conclusions of this article will be made available by the authors, without undue reservation, to any qualified researcher.

## Author Contributions

LL and YT: conceptualization. LL and LJ: data curation and analysis. FW and YT: project administration. LL, YZ, JZ, and PJ: supervision, writing—review, and editing. LL and PJ: writing—original draft.

## Funding

National Key Research and Development Program of China (2018YFC1311604 and 2016YFC1306900 to YT), National Science Fund for Distinguished Young Scholars (81725005 to Fei Wang), Liaoning Education Foundation (Pandeng Scholar to Fei Wang), Innovation Team Support Plan of Higher Education of Liaoning Province (LT2017007 to Fei Wang), Major Special Construction Plan of China Medical University (3110117059 and 3110118055 to Fei Wang), Joint fund of National Natural Science Foundation of China (U1808204 to FW), Natural Science Foundation of Liaoning Province (2019-MS-05 to FW).

## Conflict of Interest

The authors declare that the research was conducted in the absence of any commercial or financial relationships that could be construed as a potential conflict of interest.
