# Supplementary material for: The Effects of Benzodiazepine Use and Abuse on Cognition in the Elders: A Systematic Review and Meta-Analysis of Comparative Studies
Source: Front Psychiatry. 2020 Sep 17;11:00755. doi: 10.3389/fpsyt.2020.00755 (PMC7527532; doi:10.3389/fpsyt.2020.00755)
Supplement: Supplementary file 1 [file DataSheet_1.docx]

Supplementary Material

# Supplementary Table

| Research | Participant Selection | Sample Size | Confounding Variables | Statistical Analysis | Missing Data | Outcome Measures | Objective Assessment | General  assessment |
| --- | --- | --- | --- | --- | --- | --- | --- | --- |
|  | (Selection Bias) | (Performance Bias) | (Performance Bias) | (Detection Bias) | (Detection Bias) | (Reporting Bias) | (Reporting Bias) | (Total score) |
| Gray2016 | 3 | 3 | 2 | 3 | 3 | 2 | 3 | 19 |
| Hanlon1998 | 3 | 3 | 2 | 2 | 3 | 2 | 3 | 18 |
| Paterniti2002 | 2 | 3 | 2 | 2 | 3 | 3 | 3 | 18 |
| Bierman2007 | 3 | 3 | 2 | 2 | 1 | 3 | 3 | 17 |
| Mura2013 | 3 | 3 | 3 | 3 | 0 | 3 | 3 | 18 |
| Zhang2016 | 3 | 3 | 1 | 3 | 3 | 2 | 3 | 18 |
| RosCucurull2018 | 1 | 1 | 2 | 2 | 3 | 3 | 3 | 15 |
| Høiseth2013 | 1 | 2 | 2 | 3 | 3 | 3 | 3 | 17 |
| Helmes2015 | 3 | 3 | 1 | 3 | 3 | 3 | 3 | 19 |
| van Vliet2009 | 2 | 3 | 1 | 2 | 2 | 3 | 3 | 16 |
| Puustinen 2007 | 1 | 1 | 2 | 3 | 1 | 2 | 3 | 13 |
| Hessmann2019 | 1 | 2 | 2 | 3 | 3 | 2 | 3 | 16 |
| del Ser 2019 | 2 | 3 | 2 | 3 | 2 | 3 | 3 | 18 |

**Table S1.** Risk of bias ratings for the included studies.

Note: 0=Definitely no (high risk of bias); 1=Mostly no (moderate risk of bias); 2=Mostly yes (moderate risk of bias); 3=Definitely yes (low risk of bias)

#
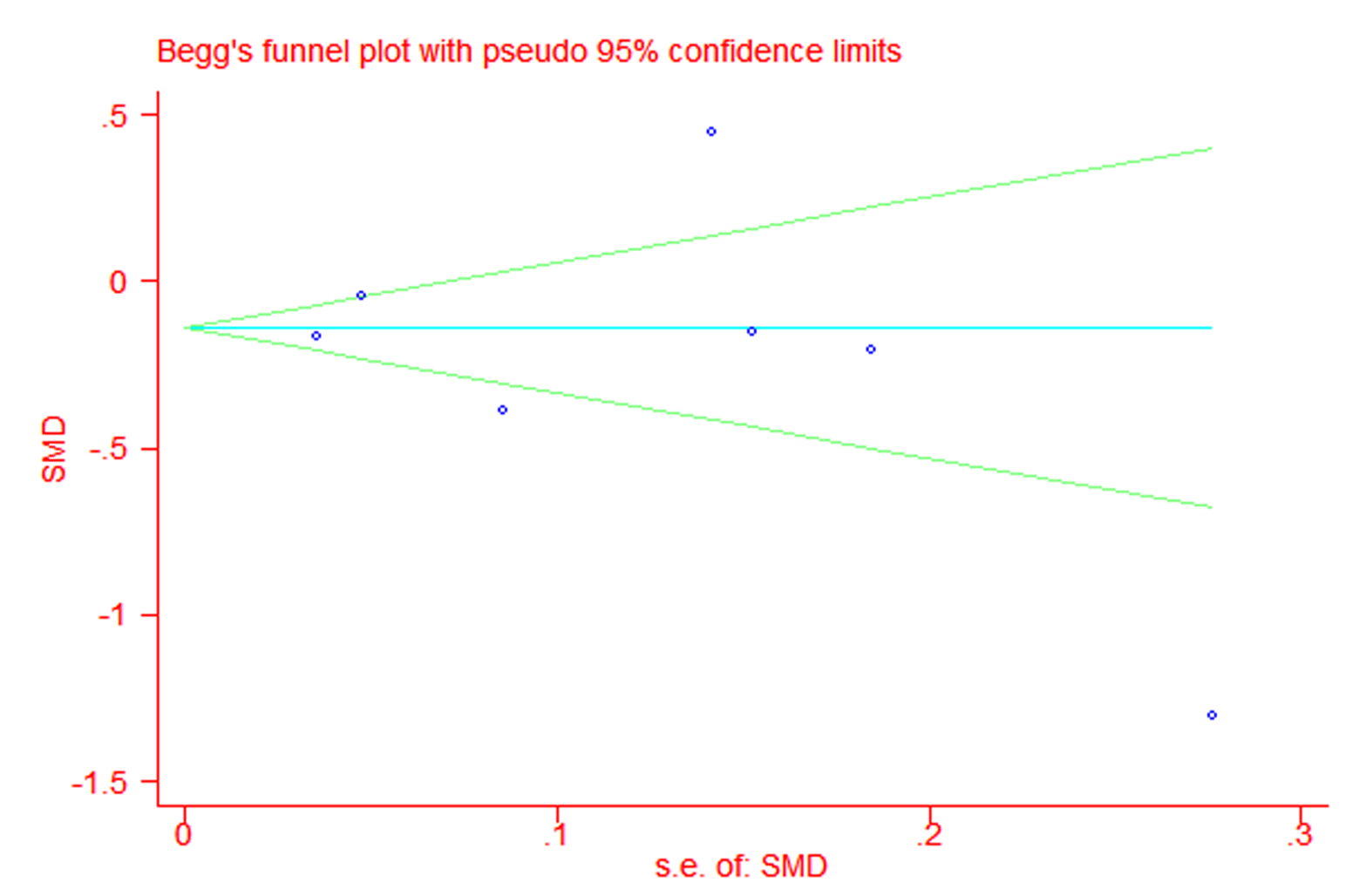
Supplementary Figure

Figure S1 Publication bias of studies evaluating Mini Mental State Examination in the elderly: Funnel plot
